# Sodium-Alginate-Functionalized Silver Nanoparticles for Colorimetric Detection of Dimethoate

**DOI:** 10.3390/bios12121086

**Published:** 2022-11-28

**Authors:** Feng-Zuo Zhou, Yung-Hsiang Chang, Cho-Chun Hu, Tai-Chia Chiu

**Affiliations:** 1 Department of Applied Science, National Taitung University, Taitung 950309, Taiwan; 2 Institute of Biochemical and Biomedical Engineering, National Taipei University of Technology, Taipei 106344, Taiwan

**Keywords:** colorimetric assay, dimethoate, silver nanoparticles, sodium alginate, water samples

## Abstract

Sodium alginate (SA) was used to functionalize the surfaces of silver nanoparticles (AgNPs) to form SA-AgNPs for sensing dimethoate with a rapid and sensitive visual readout. UV–Vis spectrophotometry, Fourier transform infrared spectroscopy, transmission electron microscopy, X-ray photoelectron spectroscopy, and zeta potential measurements were used to characterize SA-AgNPs that were synthesized under the ideal conditions. SA-AgNPs were spherical with an average size of 14.6 nm. The stability of SA-AgNPs was investigated with changes in pH, salinity, and storage time. This colorimetric assay of dimethoate relied on the change in the absorption ratio (A_475_/A_400_) of SA-AgNPs, resulting in their aggregation caused by dimethoate, leading to a visual change for SA-AgNPs from yellow to pale yellow. As a result, the absorption ratio (A_475_/A_400_) of SA-AgNPs showed good linearity in the range of 0.05 to 2.0 ppm (R^2^ = 0.9986) with a limit of detection (LOD) of 30 ppb. Adding other pesticides did not significantly change the absorption ratio of SA-AgNPs, indicating its high selectivity as a colorimetric assay. The sensor was successfully used to detect dimethoate in actual water samples.

## 1. Introduction

In modern agriculture, organophosphorus pesticides have been extensively applied to control diseases and insect pests in crops because of their low cost, low persistence, and biodegradation [1,2]. However, the excessive or improper use of organophosphorus pesticides results in high amounts of pesticides and their residues in the environment, which may harm organisms and human health [2]. Dimethoate (*O*,*O*-dimethyl *S*-[2-(methylamino)-2-oxoethyl] phosphorodithioate) is an efficient organophosphorus insecticide, and it acts by inhibiting the activity of acetylcholinesterase and disturbing the normal function of the central nervous system [3,4]. The widespread application of dimethoate in agriculture, homes, and gardens has led to its high-level residuals accumulating in the soil, surface and groundwater, and food chain. These chemicals may cause serious problems, such as fetal health hazards, birth defects, and even death [5,6]. Thus, developing a rapid, facile, and cost-effective approach for monitoring dimethoate levels is important to protect human life and prevent the ecosystem from being destroyed.

Various conventional approaches have been reported for detecting and identifying pesticides and their residues, including high-performance liquid chromatography combined with mass spectrometry, gas chromatography coupled with mass spectrometry, immunoassays, and molecularly imprinted polymer-based sensors [7,8,9,10]. Although these approaches exhibit high accuracy and sensitivity, most require time-consuming sample pretreatment procedures, expensive, bulky equipment, and experienced operators. Therefore, simple, rapid, low-cost approaches are still needed to facilitate routine pesticide analysis more conveniently. Colorimetric approaches are simple, cost-effective, and highly selective for determining pesticides compared to other approaches [11,12,13,14]. The obvious color changes in the sensing probes enable direct observation with the naked eyes upon the addition of target analytes. Recently, silver nanoparticles (AgNPs) and gold nanoparticles (AuNPs) have been used as colorimetric probes for sensing various analytes due to their unique optical and electronic properties [15,16,17]. AgNPs possess high extinction coefficients and strongly localized surface plasmon resonances in the visible region, which are very sensitive to the size, shape, and surrounding chemical environment [15]. Thus, AgNPs functionalized with various molecules can be used as colorimetric sensors and have been successfully used for detecting various target pesticides [18,19,20,21,22,23]. The sensing principle depends on the dramatic color change by the aggregation of AgNPs. For instance, Hong et al. [18] have designed colorimetric sensing of thiram using various surface capping agents, such as polyhexamethylene biguanide hydrochloride, polyvinylpyrrolidone, and borohydride ions on the AgNPs, with the LOD of 36 nM; and Dhavle et al. [19] prepared glutathione-lactose functionalized AgNPs to detect thiram with the LOD of 3.0 nM. Su et al. [20] have shown that fluorescein functionalized AgNPs for colorimetric detection of tricyclazole, and the LOD was down to 0.051 ppm. Chadha et al. [21] developed a colorimetric and Raman spectroscopy sensor for detecting trace chlorpyrifos using γ-cyclodextrin-capped AgNPs. Graphene quantum dots have been used to cap on the AgNPs and as sensors for sensing glyphosate [22] and parathion methyl [23]. Chen et al. [24] developed a rapid colorimetric detection of terbuthylazine and dimethoate based on citrate-stabilized AuNPs. The LOD for dimethoate was down to 6.2 nM. Li et al. [25] demonstrated a dual-channel localized surface plasmon resonance system based on the adsorption bands of AuNPs via optical fibers to detect dmethoate. The LOD was 5.5 nM. Although these two methods exhibit high sensitivity, their linear ranges are limited. Thus, development of a high-selectivity, high-sensitivity, linear-range assay for detecting dimethoate is needed.

**Scheme 1:**
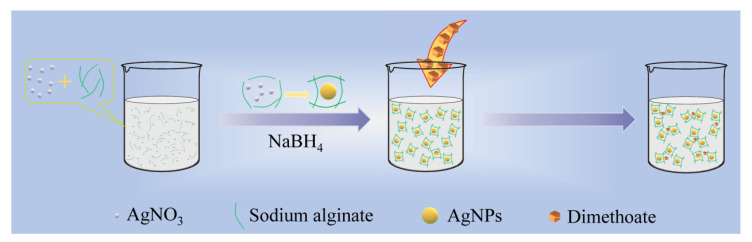
Preparation of sodium-alginate-functionalized AgNPs and their application for the colorimetric detection of dimethoate.

Sodium alginate (SA) is a naturally linear anionic carbohydrate polymer with many carboxyl groups. It is highly biocompatible and has high hydrophilicity [26]. According to previous literature [27], the carboxyl groups on the surfaces of the nanoparticles can be used to improve the stability of the nanoparticles. Owing to the electrostatic repulsion of the alginate on the surfaces of AgNPs, SA-AgNPs were well dispersed in the aqueous solution. Thus, a simple colorimetric approach based on the SA-functionalized AgNPs (SA-AgNPs) was developed for the selective determination of dimethoate. AgNPs were prepared by reducing silver salt using sodium borohydride (NaBH_4_) as a reducing agent and functionalized with SA. It is observed that only dimethoate with SA-AgNPs showed the spectral change due to the aggregation of nanoparticles caused by the interactions between dimethoate and SA-AgNPs (Scheme 1). The resulting color changes of SA-AgNPs in the absence and presence of dimethoate were further confirmed using UV–visible (UV–vis) absorption spectrometry, Fourier transform infrared (FTIR) spectrometry, transmission electron microscopy (TEM), X-ray photoelectron spectroscopy (XPS), and zeta potential measurements. The parameters such as buffer pH and reaction time were optimized to obtain improved results for determining dimethoate. Using SA-AgNPs as colorimetric probes, the current assay exhibits high selectivity, a wide linear range (0.05 to 2.0 ppm), and sensitivity to dimethoate, having a LOD of 30 ppb. Subsequently, the approach was tested in actual samples with satisfactory recoveries.

## 2. Materials and Methods

### 2.1. Chemicals and Instruments

All chemicals were of analytical grade and were used as received without further purification. Silver nitrate (AgNO_3_) was bought from Acros Organics (Geel, Belgium). 2,4-D(Sodium), acetamiprid, bifenthrin, carbaryl, carbendazim, carbofuran, chlorothalonil, chlorpyrifos, dichlorvos, dicofol, dimethoate, fenvalerate, glufosinate-ammonium, glyphosate, imidacloprid, kresoxim-methyl, methomyl, pencycuron, profenofos, propanil, trichlorfon, thiodicarb, and NaBH_4_ were purchased from Sigma-Aldrich (St. Louis, MO, USA). Sodium alginate was bought from Echo Chemical Co., Ltd. (Miaoli, Taiwan). Hydrochloric acid (HCl) was bought from Aencore (Surrey Hills, Australia). Tris(hydroxymethyl) aminomethane (Tris) was purchased from J.T. Baker (Phillipsburg, NJ, USA). The preparation of 100 mM Tris-HCl buffer dissolved 0.6067 g Tris in 50 mL deionized (DI) water and adjusted its pH from 5.0 to 9.0 with 1.0 M HCl.

UV–vis measurements were conducted on an Analytikjena Specord 210 Plus (Analytik Jena, Jena, Germany). Chemical bonding in the samples was identified using FTIR analysis via the KBr pellet method with a Nicolet iS5 spectrometer (Thermo Fisher Scientific, Waltham, MA, USA) in the mid-IR range (500–4000 cm^−1^). TEM images were obtained using a JEM-2100 transmission electron microscope (JEOL, Tokyo, Japan). Zeta potential measurements were collected using a Zetasizer Nano ZS90 particle size analyzer (Malvern Panalytical, Malvern, UK). XPS was conducted using an ESCALAB 250 X-ray photoelectron spectrometer (Thermo Fisher Scientific, Waltham, MA, USA) to analyze the chemical states of the species present and elemental composition.

### 2.2. Preparation of Sodium Alginate Functionalized AgNPs

Sodium-alginate-functionalized AgNPs (SA-AgNPs) were prepared according to our previously reported approach with minor modifications [20]. Briefly, 10 mM AgNO_3_ (250 μL) and 0.054, 0.270, 0.540, 2.701, and 5.403 mg/mL sodium alginate (250 μL) were dissolved in DI water (10 mL). Then, freshly prepared 2.5 mM NaBH_4_ (6 mL) in DI water was rapidly added to the solution under vigorous stirring at room temperature. In this study, the silver nitrate was reduced to AgNPs by NaBH_4_, and sodium alginate was used as a stabilizing agent. The color of the solution changed from colorless to bright yellow immediately after adding NaBH_4_, signifying the formation of uniformly dispersed colloidal SA-AgNPs. The SA-AgNPs were then utilized directly in subsequent studies without being purified.

### 2.3. Colorimetric Detection of Dimethoate

DI water (600 μL), 0.1 M Tris-HCl buffer (200 μL, pH 7.0), SA-AgNPs (1000 μL), and various concentrations (0.05–10 ppm) of dimethoate (200 μL) were added to a 2 mL centrifuge tube to investigate the detection ability of dimethoate. The mixture was vigorously stirred for 40 min. The resulting solution experienced a color change obvious to the naked eye, and the corresponding UV–vis absorption spectra were scanned within the 200–900 nm range. The absorption ratio at 475 to 400 nm was directly proportional to the concentration of dimethoate and was used as the analytical signal.

### 2.4. Determination of Dimethoate in Water Samples

The capability of the detecting system for actual sample analysis was tested by the determination of dimethoate in water samples. For this purpose, three concentrations (0.5, 1.0, and 1.5 ppm) of dimethoate were spiked in the drinking water samples. All water samples were filtered with 0.22 μm membranes and stored at 4 °C. The detection procedures were the same as in Section 2.3, and all experiments were repeated in triplicate to minimize experimental errors.

## 3. Results and Discussion

### 3.1. Characterization of SA-AgNPs in the Absence and Presence of Dimethoate

**Figure 1:**
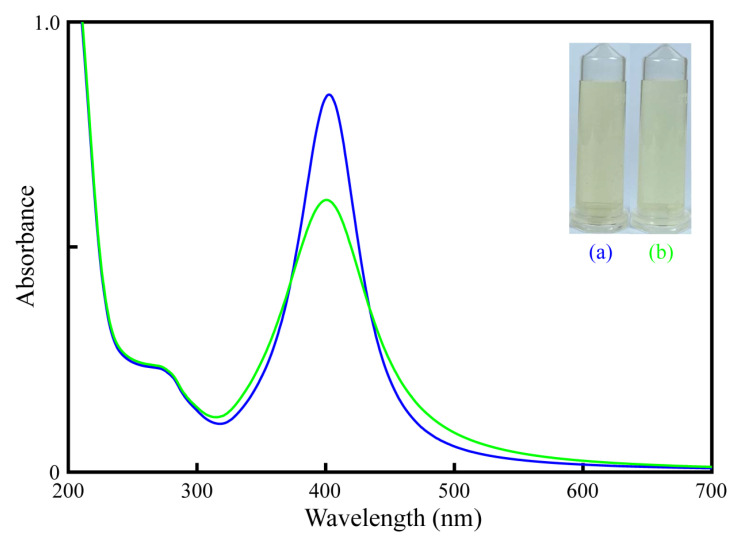
UV–vis spectra of SA-AgNPs solution (blue line) and SA-AgNPs with 10 ppm dimethoate (green line). Inset: corresponding images of (a) SA-AgNPs and (b) SA-AgNPs with dimethoate.

First, a color change from colorless to yellow confirmed the formation of the SA-AgNPs. Then, using UV–vis spectroscopy, the synthesized SA-AgNPs were characterized. The monodispersed SA-AgNPs exhibited a single characteristic absorption band at 400 nm, which is attributed to the surface plasmon resonance effect of AgNPs and shows a yellow color (inset of Figure 1). The collective oscillation of the metal-free electrons for the nanoparticle lattice in resonance with the electromagnetic source was caused by the intense interaction of incident light with metal nanoparticles [28,29]. This phenomenon is known as the SPR. The UV–vis absorption spectrum of SA-AgNPs in the presence of dimethoate is displayed in Figure 1 to investigate the feasibility of the colorimetric detection of dimethoate. It can be seen that adding dimethoate to SA-AgNPs results in a great decrease in the absorption peak at 400 nm and a slight increase in the absorption wavelength at 475 nm, revealing that dimethoate induces the aggregation of SA-AgNPs. Thus, SA-AgNPs can be fabricated to be colorimetric probes for sensing dimethoate.

**Figure 2:**
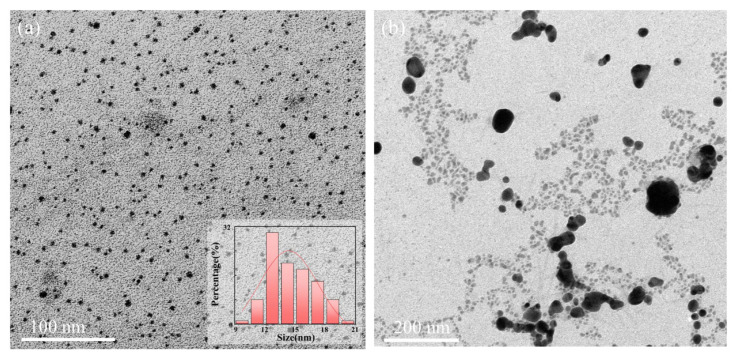
TEM images of (**a**) SA-AgNPs and (**b**) SA-AgNPs with 10 ppm dimethoate. Inset showing the particle size distribution of SA-AgNPs.

**Figure 3:**
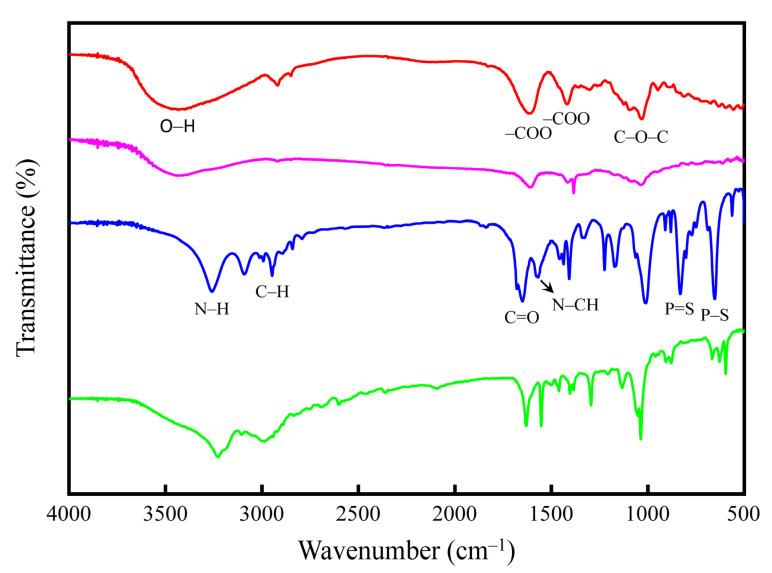
FT-IR spectra of SA (red line), SA-AgNPs (purple line), dimethoate (blue line), and SA-AgNPs with dimethoate (green line).

Figure 2 shows the representative TEM images of SA-AgNPs and SA-AgNPs in the presence of 10 ppm dimethoate. SA-AgNPs exhibited uniform spheroidal and monodisperse morphology in the TEM image. The size distribution of SA-AgNPs in diameter was 9.0–21.0 nm, and the average size was 14.6 nm. In the presence of dimethoate, the TEM image shows aggregated SA-AgNPs, and the average size of SA-AgNPs is larger than that of monodispersed SA-AgNPs. FTIR spectra of SA, SA-AgNPs, dimethoate, and SA-AgNPs with dimethoate are shown in Figure 3. A broad band centered at 3450 cm^−1^ is due to the stretching vibration band of the OH group [30]. The absorption bands at 1618 and 1420 cm^−1^ are attributed to the asymmetric and symmetric –COO stretching vibrations, respectively. The observed band at 1031 cm^−1^ can be assigned to the C–O–C stretching vibration [31]. This confirms the two main functional groups (–COO and C–O–C) in the SA molecules. SA-AgNPs contain vibrational modes consistent with the functional groups in SA, with absorption bands consistent with the two main vibrations compared with the FTIR spectra. These results suggest that the SA molecules are adsorbed on the surfaces of AgNPs. Dimethoate shows the absorption bands at 3260, 2947, 1649, 1569, 832, and 654 cm^−1^, representing the stretching vibrations of N–H, C–H, C=O, N–CH, P=S, and P–S groups, respectively [32,33]. After adding dimethoate to SA-AgNPs, the intensity and sharpness of –COO, P=S, and P–S stretching vibrations of dimethoate decreased. These spectral changes are the results of the interactions between the functional groups of dimethoate with the surface of AgNPs.

**Figure 4:**
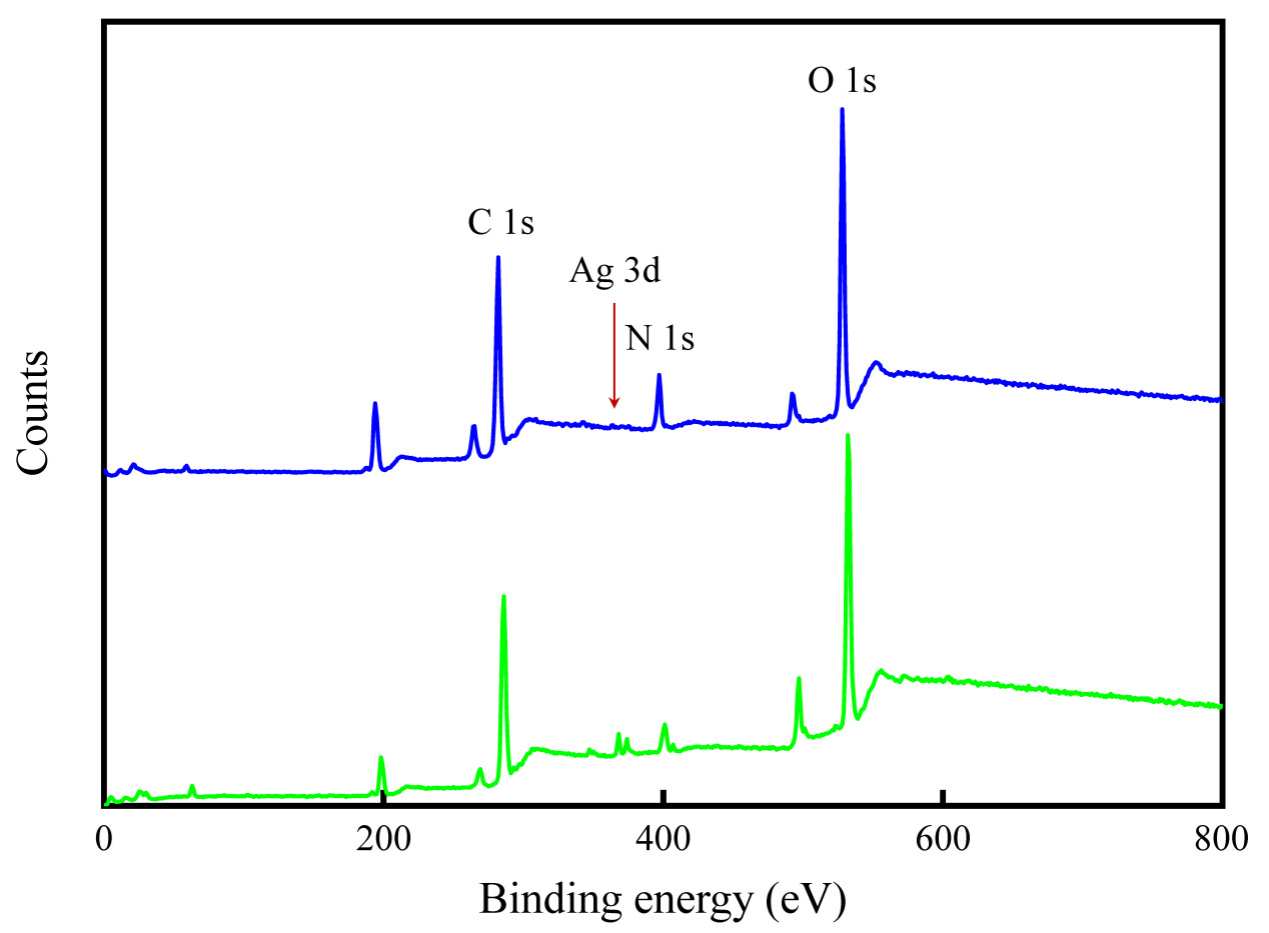
XPS survey spectra of SA-AgNPs (blue line) and SA-AgNPs with 10 ppm dimethoate (green line).

XPS was performed to investigate the chemical compositions, bonding environments, and electronic states of SA-AgNPs in the absence and presence of dimethoate (Figure 4). The binding energies at 284.8, 368.2, 400, and 531.0 eV are assigned to C 1s, Ag 3d, N 1s, and O 1s, respectively, which denotes the presence of carbon, silver, nitrogen, and oxygen. Further, the XPS spectra were recorded for SA-AgNPs after treatment with 10 ppm dimethoate. It contains characteristic peaks at 130.2, 163.9, 284.8, 368.2, 400.0, and 531.0 eV for P 2p, S 2p, C 1s, Ag 3d, N 1s, and O 1s regions, respectively. Their atomic percentages are summarized in Table S1. The high-resolution spectra of Ag 3d were measured to analyze the electronic state of AgNPs in the absence and presence of dimethoate. Two peaks associated with the spin–orbit doublets at 367.7 eV (Ag 3d5/2) and 373.7 eV (Ag 3d3/2) with a spin energy separation of 6.0 eV (Figure S1 in Supplementary Material), which is a characteristic of zero valent metallic Ag [34]. The lower-intensity peak contribution at higher binding energy is assigned to Ag atoms at the nanoparticle surface chemically bonded to the sulfur atom of dimethoate. The two Ag 3d spin–orbit pairs are separated by about 0.3 eV.

### 3.2. Stability of SA-AgNPs

Furthermore, the stability of the as-prepared nanoparticles is considered an important analytical parameter for evaluating their potential applications. Nanoparticles without suitable functionalized molecules are easy to aggregate; hence, stabilizing agents are vital for the preparation of stabilized nanoparticles. This study used sodium alginate as a functionalized ligand to stabilize AgNPs. The experimental parameters such as the amount of SA, pH of the buffer, and ionic strength have a major effect on the stability of the SA-AgNPs. One important objective of the current study was the verification of sodium alginate as a biopolymer to stabilize AgNPs. In this regard, several amounts of SA, including 0.054, 0.270, 0.540, 2.701, and 5.403 mg/mL, were considered, and their absorption ratios (A_475_/A_400_) were characterized (Figure S2a). The absorption ratio (A_475_/A_400_) is an important parameter to confirm the non-aggregation or aggregation state of the nanoparticles in colorimetric assays. The optimal amount of SA in the SA-AgNPs solution is 0.540 mg/mL, according to the results.

The stability of SA-AgNPs was evaluated at a pH ranging from 5.0 to 9.0 (Figure S2b). The SA-AgNPs were found to be stable in pH values from 5.0 to 8.0, and the stability of SA-AgNPs slightly decreased at pH 9.0. The various NaCl concentrations (0–25 mM) were treated with SA-AgNPs to evaluate the stability of the SA-AgNPs, and changes in the absorption ratio (A_475_/A_400_) of SA-AgNPs were recorded (Figure S2c). It can be seen that SA-AgNPs were stable up to 25 mM NaCl. No obvious change in the absorption ratio of SA-AgNPs was detected during the initial 35 days of SA-AgNPs being stored at 4 °C (Figure S2d). Excellent batch-to-batch reproducibility was also achieved (Figure S3). All these results confirmed that the as-prepared SA-AgNPs were highly stable and reproducible.

### 3.3. Sensing Mechanism

The preparation of sodium-alginate-functionalized AgNPs and their application for the colorimetric detection of dimethoate are shown in Scheme 1. The prepared SA-AgNPs were highly monodispersed and considered stable. The *p*Ka of the carboxyl group of sodium alginate has been reported to be 3.38 [35]. Thus, the negative charges of alginate ions on the surface of AgNPs prevent the aggregation of the SA-AgNPs. However, adding dimethoate to SA-AgNPs induces the aggregation of the SA-AgNPs and causes a color change at the optimal Tris-HCl buffer (pH 7.0). The aggregation phenomenon is due to hydrogen bonding and electron donor–acceptor interactions between dimethoate molecules and SA-AgNPs. At the optimal conditions, the hydrogen bonds can be simultaneously formed between dimethoate and alginate on the surfaces of AgNPs through –NH and –C=O of dimethoate with –COOH of alginate [36]. The electron donor–acceptor interactions could also be observed between electron-withdrawing carboxyl groups of alginate on the surfaces of AgNPs and electron-donating (N–H and P=S) groups of dimethoate. These interactions between dimethoate molecules and SA-AgNPs decreased the surface charge, inducing the aggregation of SA-AgNPs and a color change. In addition, the zeta potential measurements of SA-AgNPs and SA-AgNPs with dimethoate were −26.0 and −17.4 mV (Figure S4), further confirming the interactions between the dimethoate and SA-AgNPs. The experimental results proved the strong interactions between dimethoate and SA-AgNPs through the hydrogen bonding and electron donor–acceptor interactions.

### 3.4. Optimization of Reaction Conditions

Two analytical parameters, the pH of the solution and incubation time of SA-AgNPs with dimethoate, were optimized according to the sensing effect of the SA-AgNPs-based colorimetric assay for the determination of dimethoate. The absorption ratios of A_475_/A_400_ for the SA-AgNPs were used to optimize conditions. The results are shown in Figure S5. The pH range of the Tris-HCl buffer was adjusted from 5.0 to 9.0 to obtain the optimum condition for sensing dimethoate. It could be seen that the absorption ratios at different pH values exhibited similar outcomes. In addition, the reaction time-dependent absorption ratio of SA-AgNPs with dimethoate (1 ppm) at A_475_/A_400_ was also investigated. As depicted in Figure S5b, the absorption ratio increased in time from 0 to 40 min until it reached a plateau. The absorption ratio had almost no change from 40 to 120 min, indicating that dimethoate completely induced the aggregation of SA-AgNPs. Therefore, based on these observations, pH 7.0 and 40 min were chosen as the optimal conditions for sensing dimethoate and used throughout the experiments.

### 3.5. Selectivity, Interference, and Sensitivity of the Assay

**Figure 5:**
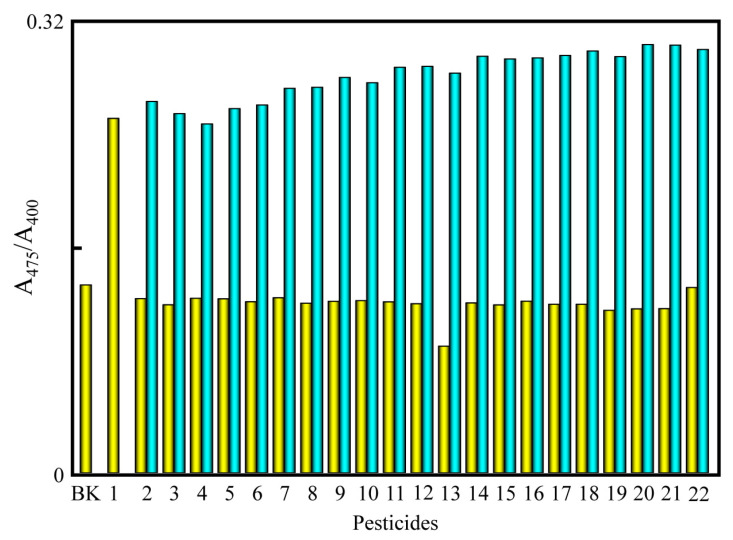
Absorption ratio (A_475_/A_400_) of SA-AgNPs for dimethoate over other pesticides (yellow bar) and the mixture of dimethoate with other pesticides (blue bar). BK: background; 1 to 22 are various pesticides (i.e., dimethoate, trichlorfon, dichlorvos, chlorothalonil, chlorpyrifos, glufosinate-ammonium, methomyl, 2,4-D(sodium), propanil, glyphosate, dicofol, carbaryl, fenvalerate, thiodicarb, acetamiprid, kresoxim-methyl, carbofuran, pencycuron, profenofos, imidacloprid, bifenthrin, carbendazim).

The selectivity of the colorimetric assay was evaluated for dimethoate compared with other interfering pesticides. Different types of pesticides, such as dimethoate, trichlorfon, dichlorvos, chlorothalonil, chlorpyrifos, glufosinate-ammonium, methomyl, 2,4-D(Sodium), propanil, glyphosate, dicofol, carbaryl, fenvalerate, thiodicarb, acetamiprid, kresoxim-methyl, carbofuran, pencycuron, profenofos, imidacloprid, bifenthrin, and carbendazim were added to the SA-AgNPs, to final concentrations of 1.0 ppm. Another 21 pesticides had no obvious effect on the UV–vis absorption spectra of the SA-AgNPs, indicating that only dimethoate can result in obvious absorption ratio (A_475_/A_400_) changes to the SA-AgNPs, as shown in Figure 5. The results imply excellent selectivity of this colorimetric assay for detecting dimethoate. Additionally, to further verify the feasibility of the SA-AgNPs for the recognition of dimethoate, potential interference from other pesticides (1.0 ppm) was recorded (Figure 5). According to the data, other pesticides cause very slight changes in the absorption ratio (A_475_/A_400_). To further confirm the practical application, the interference tests of the SA-AgNPs towards dimethoate were performed with lake water samples. As shown in Figure S6, only a slight change in absorption ratio (A_475_/A_400_) was observed. These results show that other pesticides cause no interference with dimethoate detection.

**Figure 6:**
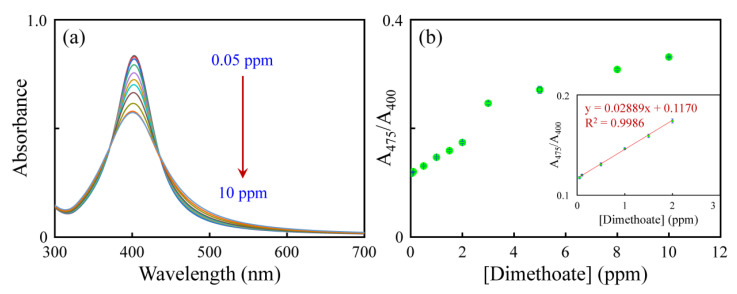
(**a**) UV–vis absorption spectra and (**b**) linear response of the absorption ratio at A_475_/A_400_ plotted against the dimethoate concentration (*n* = 3). Inset: a linear calibration curve between A_475_/A_400_ and the concentration of dimethoate (0.05–2.0 ppm).

Various concentrations of dimethoate ranging from 0 to 10.0 ppm were examined under optimal conditions to show the performance of the sensing system. UV–vis absorption spectroscopy monitored the variations in colorimetric response at the absorption ratio (A_475_/A_400_) of SA-AgNPs upon adding dimethoate. The concentration of dimethoate increases and the absorption ratio (A_475_/A_400_) of SA-AgNPs increases over a wide range of concentrations (0.05–2.0 ppm), as shown in the inset of Figure 6. The change in absorption ratio (A_475_/A_400_) plot against the dimethoate concentration from 0.05 to 2.0 ppm gave a straight line with R^2^ = 0.9983. The LOD of dimethoate was calculated from the standard deviation (σ) of the blank (*n* = 10) and slope (s) of the calibration curve using the following equation [37]:

LOD = 3σ/s(1)

The calculated LOD of dimethoate was 30 ppb.

Table 1 compares the analytical performances of the developed colorimetric assay for dimethoate based on the aggregation of SA-AgNPs to other reported assays. Although some assays exhibit lower LODs, their linear ranges are limited. The proposed method has higher selectivity for detecting dimethoate than other assays. Thus, the results show that SA-AgNPs can be used as efficient probes for the colorimetric detection of dimethoate with a wider linear range, higher selectivity, and a comparable LOD.

### 3.6. Determination of Dimethoate in Water Samples

The drinking water samples were analyzed using SA-AgNPs as colorimetric probes to test the practicability and accuracy of the developed sensor for dimethoate detection. Under the optimal experimental conditions, SA-AgNPs were treated with 0.5, 1.0, and 1.5 ppm dimethoate in the drinking water samples. The ultimate concentrations of dimethoate could be obtained based on the changes in absorption ratio (A_475_/A_400_) and the linear regression equation of the calibration curve. Table 2 summarizes the quantitative results. The recoveries varied from 90.3% to 106.6%, and the relative standard deviation (RSD) was within 7.4%. It was observed that there were no severe interferences in drinking water samples, which revealed relatively high precision. Consequently, it was believed that the SA-AgNPs-based colorimetric assay could be successfully applied to determine dimethoate with high precision and accuracy in drinking water samples.

## 4. Conclusions

In this study, SA-AgNPs were synthesized via a chemical reduction method. The as-prepared SA-AgNPs were characterized through UV–vis, FTIR, TEM, and XPS measurements. The recognition ability of SA-AgNPs towards dimethoate was evaluated using UV–vis, FT-IR spectrometry, XPS, and zeta potential measurements. Adding dimethoate to SA-AgNPs produces a significant decrease in the absorption ratio (A_475_/A_400_) of SA-AgNPs, along with a color change. The yellow color of the SA-AgNPs changed into light yellow via the electron donor–acceptor interactions and hydrogen bonding with dimethoate molecules. The other tested pesticides did not produce an obvious change in the absorption spectra or color of the SA-AgNPs. In the presence of other pesticides, the SA-AgNPs were highly selective in recognizing the dimethoate, as no interference was observed in the competitive experiments. Furthermore, SA-AgNPs were successfully used to detect dimethoate in drinking water. The current assay’s detection limit is comparable to those of other reported methods.

## Figures and Tables

**Table 1:** Comparison of the proposed assay with other reported assays for the colorimetric detection of dimethoate.

Probes	Linear Range (ppb)	LOD (ppb)	Selectivity	Applications	Ref.
Ni(PhDP)_2_	98.5–596	91.7	3 inorganic salts	Urban, lagoon, stream, groundwater, treated wastewater	[38]
MIP-CoZn ZIF	4.59–275	1.28	11 pesticides	Orange, Lemon, agriculture wastewater	[39]
GO	2–200	2	–	–	[40]
Citrate-AuNPs	10–400	4.7	8 pesticides	Tomato, cucumber, cabbage	[41]
Citrate-AuNPs	0.23–9.2	1.42	19 pesticides	Tap water, green tea, apple juice	[24]
Citrate-AuNPs	2.29–22.93	1.26	4 pesticides	Apple	[25]
Ag_2_O NPs	20–160	14	11 pesticides	Pepper, Green beans, Cabbage	[42]
pSC_4_R-AgNPs	22.9–229	18.3	7 pesticides	Industrial waste water	[43]
Smartphone-printed-paper	100–2000	30	8 pesticides	Tomato, radish	[33]
Cu@AgNPs	50–2500	16	8 pesticides	–	[33]
AgNPs	688–4585	688	–	–	[44]
CuO NPs	688–4585	688	–	–	[44]
Ag-Cu NPs	688–4585	688	–	–	[44]
SA-AgNPs	50–2000	30	22 pesticides	Drinking water	This work

GO: graphene oxide; LOD: limit of detection; MIP: molecularly imprinted polymer; Ni(PhDP)_2_: bis 5-phenyldipyrrinate of nickel (II); NPs: nanoparticles; pSC_4_R: p-sulphonato-calix [4]resorcinarene; SA: sodium alginate; ZIF: zeolitic imidazole framework.

**Table 2:** Recoveries for detecting dimethoate with the proposed assay in drinking water samples. (*n* = 3).

Sample	Spiked (ppm)	Found (ppm)	Recovery (%)	RSD (%)
1	0.5	0.52	104.0	7.4
2	1.0	1.06	106.6	3.8
3	1.5	1.35	90.3	3.9

## Data Availability

The data supporting the findings in this research are available upon reasonable request.

## Supplementary Materials

The following supporting information can be downloaded at: https://www.mdpi.com/article/10.3390/bios12121086/s1. Figure S1: High-resolution Ag 3d XPS spectra of (a) SA-AgNPs and (b) SA-AgNPs with dimethoate. Figure S2: Effects of (a) the amount of SA, (b) pH, (c) NaCl concentrations, and (d) storage time on the absorption ratio A_475_/A_400_ of SA-AgNPs. Inset: the images of various amounts of SA stabilized AgNPs. Figure S3: The batch-to-batch reproducibility for the preparation of the SA-AgNPs. Figure S4: Zeta potentials of SA-AgNPs and SA-AgNPs with dimethoate (10 ppm). Figure S5: Effects of (a) pH and (b) incubation time on the absorption ratio of A_475_/A_400_ of SA-AgNPs with dimethoate. Figure S6: The absorption ratio (A_475_/A_400_) of the SA-AgNPs towards dimethoate with other pesticides in the lake water samples. The peak identifies are the same as in Figure 5. Table S1: XPS results of elemental analysis.
