# Supplementary material for: Sodium-Alginate-Functionalized Silver Nanoparticles for Colorimetric Detection of Dimethoate
Source: Biosensors (Basel). 2022 Nov 28;12(12):1086. doi: 10.3390/bios12121086 (PMC9775393; doi:10.3390/bios12121086)
Supplement: Supplementary file 1 [file biosensors-12-01086-s001.zip › biosensors-2025330-supplementary.pdf]

biosensors

Supplementary Materials

Sodium-Alginate-Functionalized Silver Nanoparticles for Colorimetric Detection of Dimethoate

Feng-Zuo Zhou [1], Yung-Hsiang Chang [2], Cho-Chun Hu [1] and Tai-Chia Chiu [1,*]

[1] Department of Applied Science, National Taitung University, Taitung, Taiwan
[2] Institute of Biochemical and Biomedical Engineering, National Taipei University of Technology, Taipei, Taiwan
* Correspondence: tcchiu@nttu.edu.tw; Tel.: +886-89-517-990

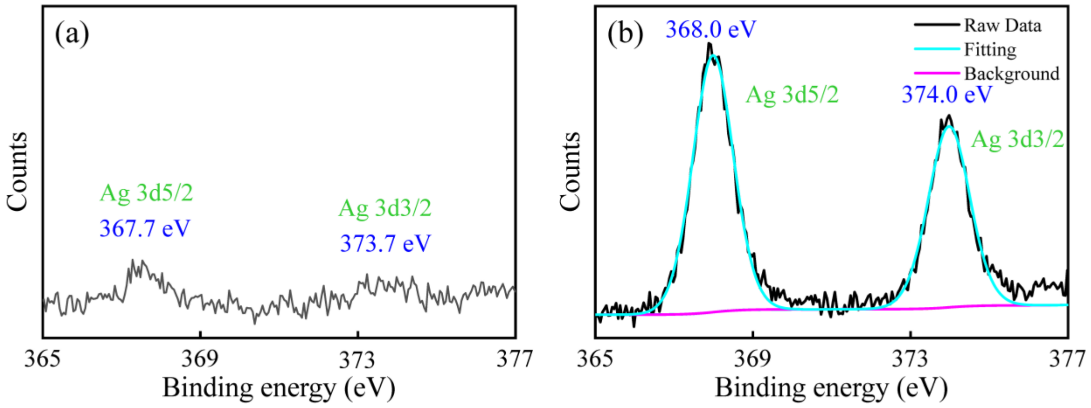

Figure S1. High-resolution Ag 3d XPS spectra of (a) SA-AgNPs and (b) SA-AgNPs with dimethoate.

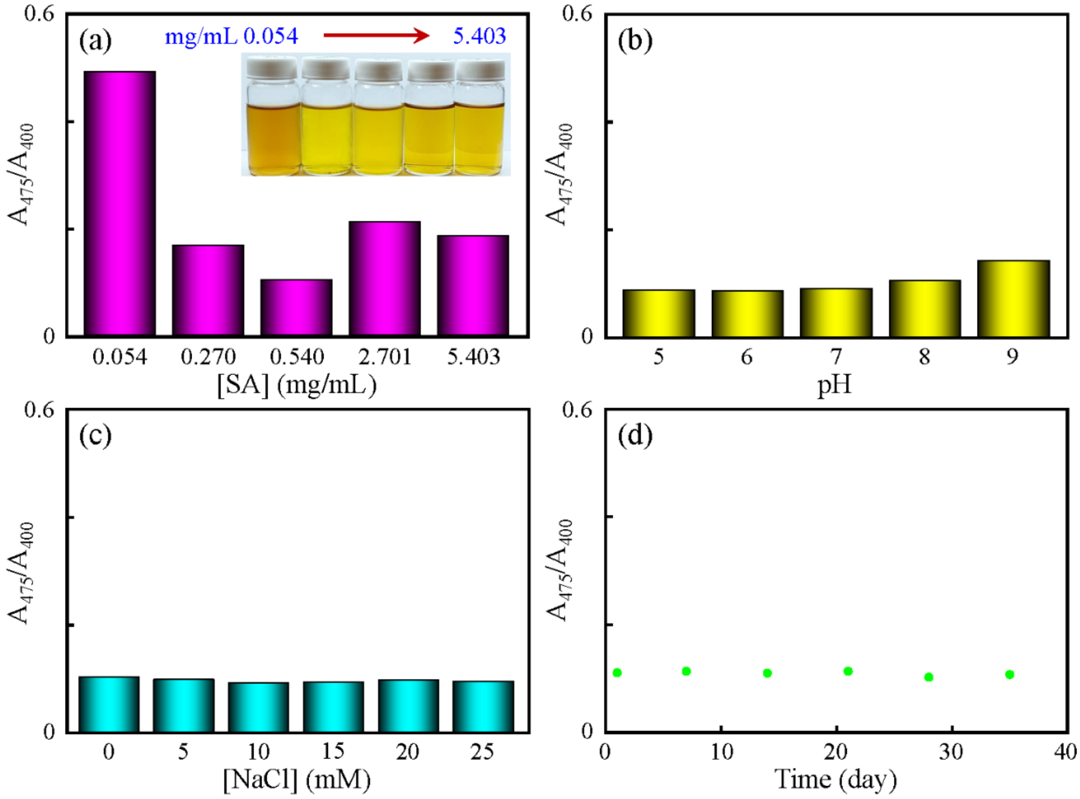

Figure S2. Effect of (a) the amount of SA, (b) pH, (c) NaCl concentrations, and (d) storage time on the absorption ratio A_{475}/A_{400} of SA-AgNPs. Inset: the images of various amounts of SA stabilized AgNPs.

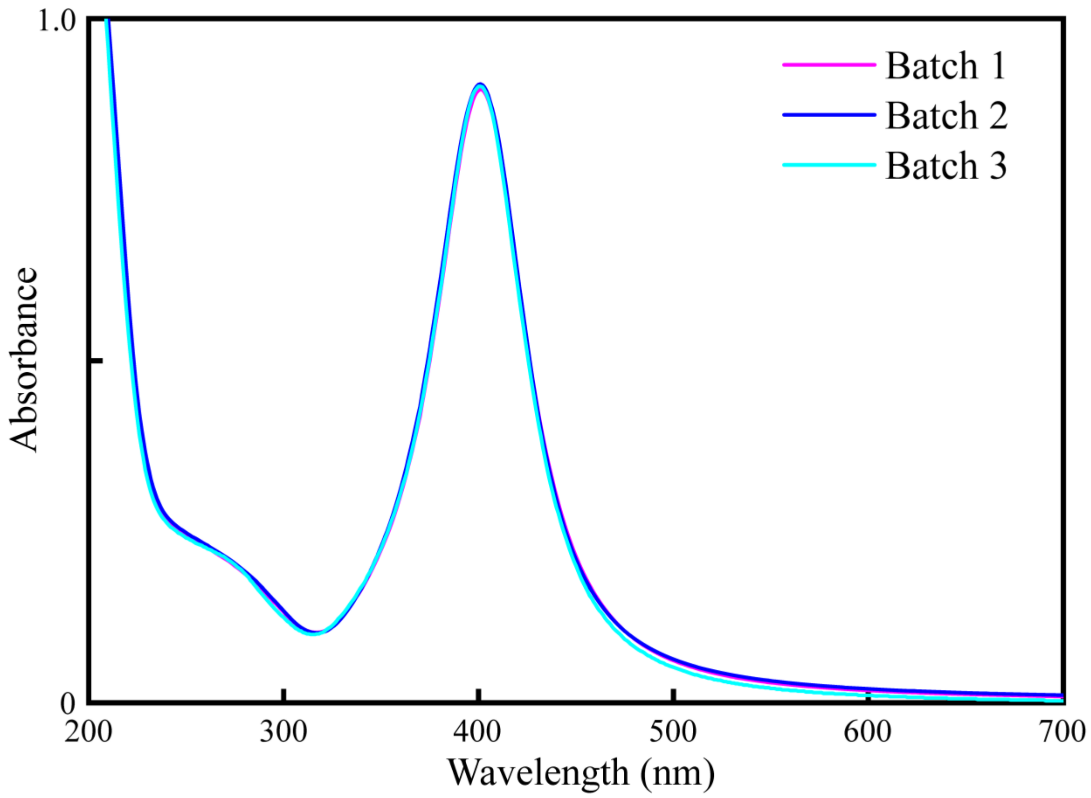

Figure S3. The batch-to-batch reproducibility for the preparation of the SA-AgNPs.

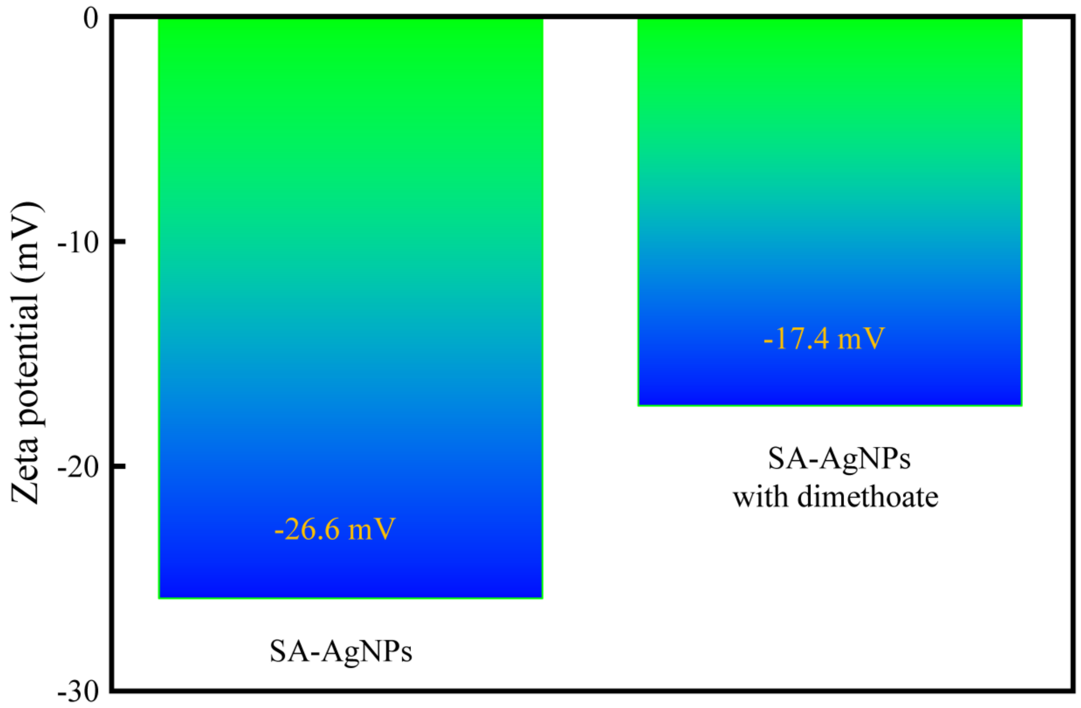

Figure S4. Zeta potentials of SA-AgNPs and SA-AgNPs with dimethoate (10 ppm).

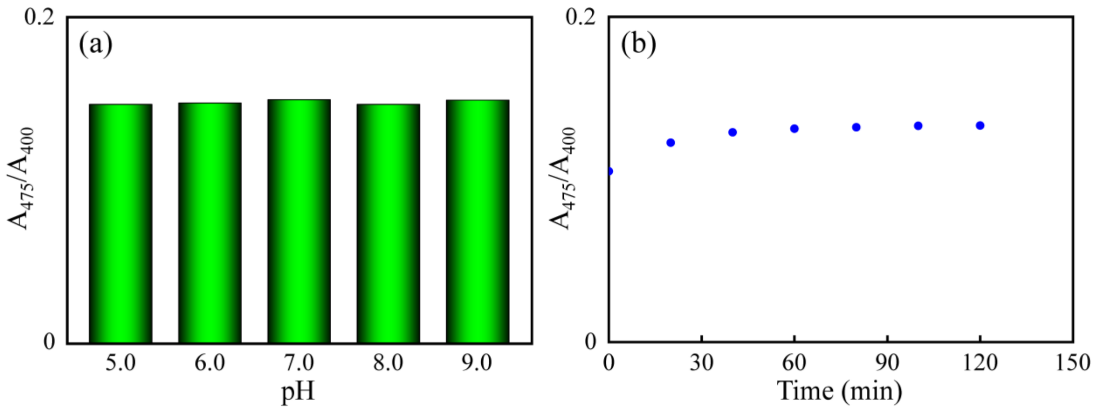

Figure S5. Effect of (a) pH and (b) incubation time on the absorption ratio of A_{475}/A_{400} of SA-AgNPs with dimethoate.

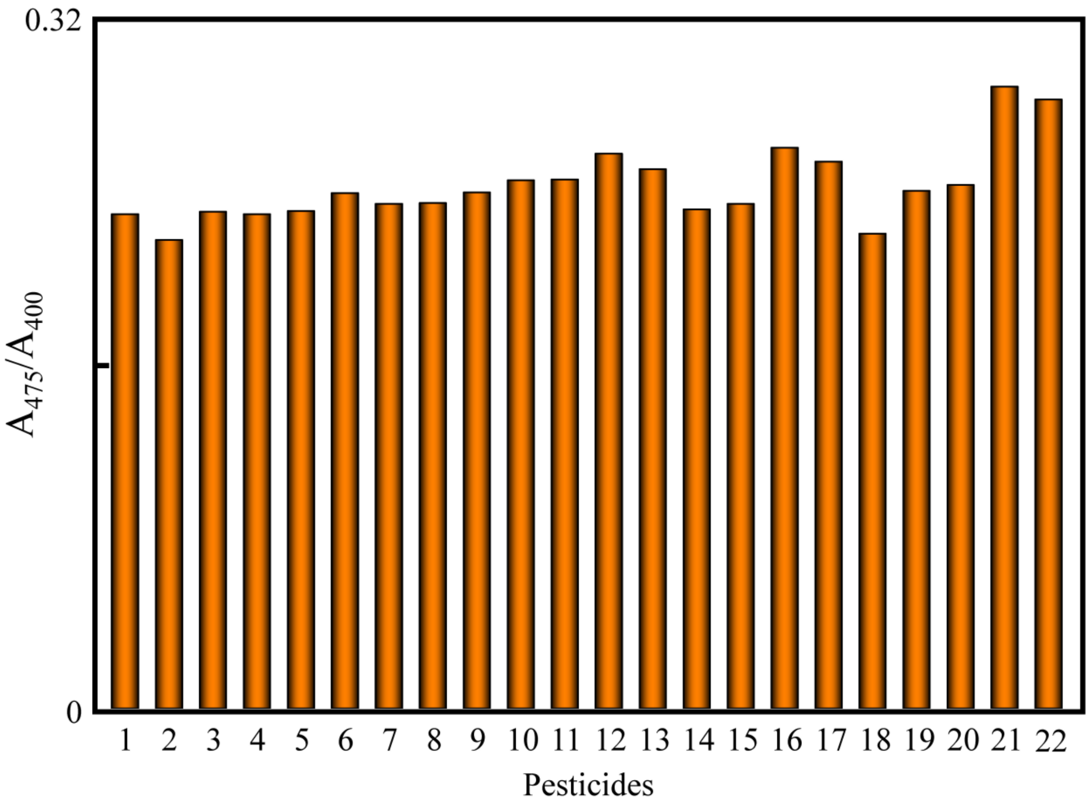

Figure S6. The absorption ratio (A_{475}/A_{400}) of the SA-AgNPs towards dimethoate with other pesticides in the lake water samples. The peak identifies are the same as Figure 5.

biosensors

MDPI

Table S1. XPS results of elemental analysis.

**Sample**	**Atomic concentration of element (%)**					
	**Ag 3d**	**C 1s**	**N 1s**	**O 1s**	S 2p	P 2P
SA-AgNPs	0.04	54.19	5.43	40.33	–	–
SA-AgNPs with dimethoate	0.11	63.82	4.61	31.15	0.17	0.13
